# Examining the impact of combining yoga-based breathing techniques with short-bouts of walking on state anxiety: findings from two pilot randomised trials

**DOI:** 10.1080/21642850.2026.2629646

**Published:** 2026-02-13

**Authors:** Kostyantyn Yaremenko, Kathryn Greenslade, Ailsa Niven, Graham Baker

**Affiliations:** a Physical Activity for Health Research Centre, The University of Edinburgh, Edinburgh, Scotland, UK; b Undergraduate Centre, Manchester Royal Infirmary, Manchester, England, UK

**Keywords:** Well-being, anxiety, yoga, walking, mental health

## Abstract

**Background:**

Single bouts of exercise can reduce state anxiety, but little is known about the benefits of short-duration walking and the potential additional benefits of integrating yoga-based breathing techniques. This study aimed to investigate whether performing a yoga-based breathing pattern, breathwalk (BW), offers additional acute benefits compared to normal breathing, on state anxiety during a single bout of walking.

**Methods:**

Two pilot randomised trials (parallel groups) were conducted in healthy adults (*n* = 37 and *n* = 47) in different naturalistic settings. In both trials, participants (students/staff in one University) were randomly assigned to the breathwalk (BW) or normal walking (NW) group. All the participants performed one short bout of walking at a self-selected moderate pace. The BW group also followed a breathwalk protocol of step-based inhalation and exhalation during the walking bout. The outcome measure in both trials was state anxiety (State Trait Anxiety Inventory for Adults™) assessed pre- and post-walking bout. Participants and outcome assessors were not blinded. Analyses were performed using two-way mixed factorial analysis of variance with follow-up paired sample t tests. Cohen's *d* effect sizes provided an indication of the magnitude of change within each group.

**Results:**

In trial 1, there was no significant (group × time) interaction, but a significant main effect represented a reduction in state anxiety in both groups. In Trial 2, a significant interaction effect was found; only the BW group showed a significant reduction in anxiety over time. In both trials, effect sizes (reduction in state anxiety) were greater in the BW compared with the NW group.

**Conclusion:**

These trials provide further evidence of the beneficial effects of short, single bouts of walking on the mental well-being of young adults. There was preliminary evidence that the incorporation of a simple, breathing technique (breathwalk) provided greater anxiolytic effects than walking alone which warrants further investigation.

## Introduction

Anxiety is ‘characterised by unpleasant feelings of apprehension and thoughts of worry that are frequently combined with autonomic nervous system activation’ (Herring, 2018, *p*. 21). Anxiety is a normal evolutionary response to threat; however, anxiety can become disordered if it becomes prolonged, severe, occurs in the absence of threat, and starts to interfere with daily functioning. There has been a growth in the global prevalence of anxiety disorders by 26% due to the COVID-19 pandemic (Santomauro et al., 2021) and there is a need to effectively mitigate against and treat this condition.

Physical activity can be beneficial in protecting against the emergence of anxiety disorders (McDowell et al., 2019; Schuch et al., 2019), and in improving anxiety disorder symptoms (Ramos-Sanchez et al., 2021; Singh et al., 2023). A potential contributing mechanism for this therapeutic effect is repeated exposure to the beneficial effects of individual bouts of physical activity (Perkins et al., 2023). Indeed, a series of meta-analyses, starting with an original study in 1991 (Petruzzello et al., 1991) and subsequently updated in 2015 (Ensari et al., 2015) and 2023 (Connor et al., 2023), provide evidence for a small, reliable anxiolytic benefit of acute bouts of physical activity on state anxiety (i.e. anxiety which is acute and temporary; (Ensari et al., 2015)). These studies also report heterogeneity in findings which, despite insufficient studies to draw definitive conclusions, does indicate that a range of factors may moderate the effect. For example, there was mixed evidence relating to intensity, with Ensari and colleagues highlighting that higher intensity may be more beneficial (Ensari et al., 2015), whilst a subsequent review demonstrated beneficial effects for both moderate- and high-intensity activity (Connor et al., 2023). Furthermore, a relatively consistent finding from the reviews was that longer duration bouts of exercise (i.e. >20 min (Petruzzello et al., 1991); >30 min Connor (Connor et al., 2023)) appears to be more beneficial than shorter durations. Nevertheless, further consideration of shorter durations would be valuable to investigate the benefits of more incidental physical activity that can be easily integrated and experienced in daily living. Furthermore, the majority of the included studies were lab-based, utilising treadmills and cycle ergometers for aerobic exercise, optimising the internal validity of the studies but also reducing the external validity and applicability to lifestyle physical activity. There would be value in evaluating the acute effects of activity on state anxiety in more ecologically valid settings.

Despite the growing evidence for the benefits of physical activity on both mental and physical health; large numbers of people are insufficiently active to achieve these established health benefits (Strain et al., 2024) with global inactivity levels labelled as pandemic (Kohl et al., 2012). Identifying physical activities that are likely to be adopted on a large scale is a priority to successfully capitalise on the benefits for managing anxiety. Walking has been labelled the ‘nearest activity to perfect exercise’ (Morris & Hardman, 1997, *p*. 328), and a best buy for public and planetary health (Bull & Hardman, 2018). Walking is a mode of physical activity that is accessible to most groups of the population, can be integrated into daily living, and has been advocated as the easiest way to gain recommended levels of daily physical activity (Bull & Hardman, 2018). Importantly, walking at an average or brisk pace (≥100 steps per minute) is likely to be most beneficial for physical health (Stamatakis et al., 2018; Tudor-Locke et al., 2018).

Whilst there is growing evidence of the benefits of walking interventions on anxiety symptoms (Kelly et al., 2018; Xu et al., 2024); there is limited research considering the anxiolytic beneficial effects of single bouts of walking, with Kelly and colleagues scoping review identifying only four studies with mixed findings. Any benefits of walking for anxiety reduction may also potentially be amplified or confounded by interventions that integrate other strategies known to be beneficial. For example, deep voluntary breathing, such as that conducted in meditation or yoga, represents a behavioural practice which may independently and positively impact on relief from stress and anxiety states. Slow and deep breathing has a calming effect on the mind and helps an individual to de-stress (Bhattacharya et al., 2002). Edwards and colleagues reported that meditation and meditation plus 10 min of brisk walk (either before or after) was beneficial in significantly reducing state anxiety, but brisk walking alone was not (Edwards et al., 2018). Additionally, Shin et al. reported meditative walking led to greater reductions in state anxiety than athletic walking in both a gym and forest environment (Shin et al., 2013).

Breathwalk (BW) is a contemporary interpretation of a yoga breathing technique called *Sama-Vritti-Pranayama* (Sanskrit: ‘Equal Fluctuation of Breath’ or ‘Even Breath’) (Bentley et al., 2023) incorporated into normal walking. It is a combination of steps synchronised with a specific breathing pattern with attention concentrated on the breathing element (Khalsa, 2008). To date, there has been one published study investigating the BW technique (Vázquez-Vandyck et al., 2007). This study recruited 17 patients who followed the BW protocol three times per week for an intervention period of six months and reported beneficial effects on select metabolic outcomes (dyslipidaemia and insulin resistance). However, this study was of a single-arm design and underpowered, included only individuals diagnosed with hepatitis C virus (HCV), and did not consider anxiety outcomes. Therefore, any potential wider benefits of the BW technique on otherwise healthy individuals, and anxiety specifically have not yet been explored. Furthermore, there has been no robust consideration of the benefits of a single bout of BW.

To summarise, there is some evidence that single bouts of exercise are beneficial for state anxiety; however, there is limited research focusing on short bouts of walking in a free-living context. Additionally, to the best of our knowledge no research has considered the potential additional benefit of the BW technique. Therefore, the main aim of this study was to investigate whether performing the BW technique during a single, short bout of walking provides any additional benefit on measures of state anxiety over normal walking.

## Methods

### Study design

Two experimental studies were conducted to investigate the acute effects of the BW protocol on state anxiety during different naturalistic settings in Edinburgh, United Kingdom (UK). These were considered ‘external pilot randomised trials’ according to the definition from Eldridge and colleagues' conceptual framework (Bond et al., 2023; Eldridge et al., 2016); small-scale studies to assess the feasibility of implementing the breathwalk technique in different walking protocols and settings to address potential major issues related to intervention refinement. Technical reporting in both studies followed the Consolidated Standards of Reporting Trials (CONSORT) extension to randomised pilot and feasibility trials (Eldridge et al., 2016) (see Additional files 1 and 2). Trial protocols were reviewed and approved by relevant Moray House School of Education and Sport Ethics Subcommittee (KY000305), University of Edinburgh. Trials were not pre-registered. Both trials utilised a 2-group between-subjects, randomised parallel-group design in a 1:1 ratio. A ‘between-subjects’ design was used to eliminate any potential carry-over effect of the BW technique from a ‘within-subjects’ design. There were no changes to the methods after trial commencement.

### Participants and recruitment

#### Sample size

A sufficient number of participants for each trial was considered incorporating recommendations for pilot and feasibility studies. It was not the aim of these studies to provide data for sample size calculations for a definitive trial or include as any pre-defined progression criteria, nor to test specific hypothesis. Rather, it was to explore proof of concept of breathwalk as an intervention technique alongside a well-established mode of activity (walking) to reduce state anxiety. Therefore, these studies utilised published rule of thumb guidelines for studies of this nature aiming for between 15 and 20 participants per intervention arm (Julious, 2005; Kieser & Wassmer, 1996).

#### Inclusion and exclusion criteria

The inclusion and exclusion criteria for both trials can be found in Table 1. A key exclusion criterion in both studies was that participants should not have any previous experience with the breathwalk technique, as this may create bias in the study results.

**Table 1.** Shared and trial specific eligibility criteria.

	Inclusion criteria	Exclusion criteria
*Both trials*	Able to walk independently for at least 10 min	Smokers
	Healthy in terms of absence of chronic obstructive pulmonary disease (COPD), cardiovascular disease (CVD) and respiratory illness	Practiced in BW technique
*Trial 1*	Adult volunteers aged 19–65 years	None
*Trial 2*	University students aged 18–25	None
	Walking commute to university	
	Travel time between 10 and 30 min	

#### Recruitment

Participants in both trials were recruited via adverts (online and physical posters) from one academic department at the University of Edinburgh, social media sources and snowball sampling (Parker et al., 2019). In Trial 1, both students and staff were recruited, whilst in Trial 2 the study had a focus on improving the psychological well-being of university students and recruited from this population group only. In Trial 2, the active commute (by walking) of university students was targeted and thus an essential inclusion criterion was that students' commute from their home address to the University library (a central campus location where all students attend) be between 10 and 30 min in duration; sufficient to elicit health benefits as recommended (World Health Organisation, 2010) and representative of a typical Edinburgh commute (The City of Edinburgh Council., 2016). Following an initial expression of interest, potential participants were provided with a study information sheet. After providing informed consent and prior to baseline data collection, participants completed a medical screening questionnaire and a physical activity readiness questionnaire (PAR-Q) form (Thomas et al., 1992).

### Randomisation

In Trial 1, randomisation was conducted using a simple coin-tossing method for the first occasion and then an alternate group method to ensure balance between groups. In Trial 2, this was conducted using a Microsoft Excel programme given the additional complexity of stratification by typical walk time of ‘10–20’ or ‘20–30’ minutes to minimise selection bias. Journey time was a stratification variable due to potential variation in benefits with a longer or shorter walk time. In both trials, participants randomised to a normal walking condition were blinded to the instructions of the breathwalk condition but were provided with this information upon completion of the study. Enrolment and allocation were performed by the lead researcher of each trial (KY Trial 1; KG Trial 2).

### Interventions

In both trials, it was not possible to blind participants or those delivering the intervention or assessing outcomes to intervention allocation.

#### Trial 1

Participants were randomised to either a normal walking (NW) condition or a breathwalk (BW) condition. Both groups were instructed to undertake one single bout of walking of approximately 10 min in duration walking at a moderate pace, this bout length stated as sufficient to elicit health benefits in previous physical activity guidelines (e.g. U.S. Department of health and Human Services., 2008). The walking was performed in the green environment of a park local to the research institute. A pre-determined distance and route was created to minimise any potential bias or impact of city traffic and air pollution (Barton et al., 2009) and ensuring a flat surface to avoid the impact walking uphill and downhill can have on the ratio of inhalation and exhalation and resultingly on heart rate and blood pressure (Minetti et al., 2002). All participants were given a map with the pre-defined route. The overall route distance of 1000 m (1 km) was selected based on the distance that a person can cover using normal walking pace on a flat surface at a step-rate of approximately 100 steps/min which is indicative of moderate intensity physical activity (Abel et al., 2011).

**Figure 1.**
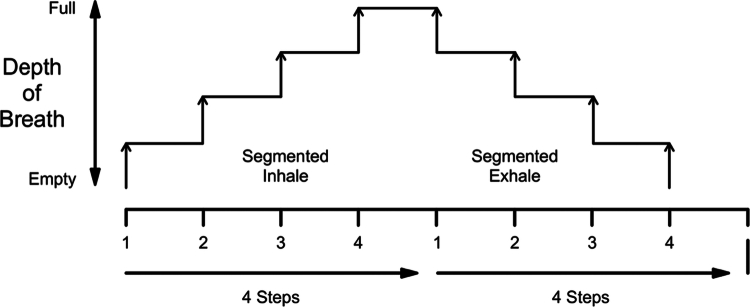
Breathwalk technique scheme (adapted from (Khalsa, 2008)).

Trial 1 was delivered by a Masters student studying physical activity for health with expertise in yoga. Participants in the BW group were provided with a guidance sheet with the BW protocol, this was also explained verbally. The breathwalk technique is a normal, moderately paced walk with looped four steps synchronised with inhale and four steps synchronised with exhale (nasal breathing; see Figure 1) and accomplished by attention directed to the body movements and breathing process (Khalsa, 2008). The breathing frequency tends to follow frequencies of steps due to increasing metabolic rate; breathing rate increases from 15 breaths per/min at rest to 40–60 breaths per/min (mean = ~50 breaths per/min) during moderate paced walking (European Lung Foundation 2016). During the trial, BW participant would have therefore have approximately 25 breaths per/min, because of the 4:4 breathing ratio (four steps whilst inhale and four steps whilst exhale; see Table 2).

**Table 2.** Combined NW and BW intervention parameters for both trials.

	Trial 1	Trial
Time	10 min	10–20 min and 20–30 min
Distance	1000 m (~1 km)	~1–2 km and ~2–3 km
Speed	Moderate pace: 1.4 m/sec (5 km per/hour)	Moderate pace: 1.4 m/sec (5 km per/hour)
Breaths	NW = ~50 breaths per/min BW = ~25 breaths per/min	NW = ~50 breaths per/min BW = ~25 breaths per/min
Type of breath	Nasal breathing	Nasal breathing

#### Trial 2

Trial 2 was delivered by a medical student studying a bachelor's degree in physical activity for health. The participants were again randomised to either a normal walking (NW) condition or a breathwalk (BW) condition. The focus of this study was to explore the potential impact of BW in a free-living setting. All the participants walked their normal route from home to the university library at a self-selected normal pace on a single occasion. This creates ecological validity by representing a real walking route rather than being artificially constructed (Andrade, 2018). To minimise confounding factors that may influence walking pace and breathing rate, participants were asked not to listen to music or use their mobile phone whilst walking. The process and content of BW technique instructions and technique were consistent with those described in Trial 1 (see Table 2 for the intervention parameters in both trials).

### Outcomes

#### Anxiety

The primary outcome measure of state anxiety in both trials was assessed using the STAI-AD form (State-Trait Anxiety Inventory for Adults™, Mind Garden, Inc. (Spielberger et al., 1983), which includes 20 items measuring state anxiety. Participants rate how they feel themselves ‘right now, at this moment’ using a Likert-type scale ranging from 1 (not at all) to 4 (very much so) on feelings of tension, worry, apprehension, nervousness and arousal of the autonomous nervous system. Nine items represent calm conditions (e.g. ‘I feel calm’, ‘I feel secure’) and eleven items represent anxiety conditions (e.g. ‘I feel tense’, ‘I feel nervous’). Non-anxiety items are then reverse scored (Spielberger et al., 1983). Participants completed the measure immediately pre and post walking bout. No changes to assessments were made following commencements of the trials.

#### Perceived exertion

The Borg Rating of Perceived Exertion scale (RPE) was used to assess perceptions of exertion after walking for both trials to examine physiological equivalence between intervention conditions. The scale stems from the linear interdependence of heart rate (HR), stimulus intensity and oxygen consumption and correlates with a HR of 60 and 200 beats, respectively in healthy adults (Borg & Kaijser, 2006). Thus, the RPE instrument with scale from 6 (no exertion at all) to 20 (maximal exertion) was used in this study (Borg & Kaijser, 2006) and completed immediately post walk only. No changes to assessments were made following commencements of the trials.

### Statistical analysis

Data in both trials were analysed using the Statistical Package for the Social Sciences software, Versions 24 and Version 25, respectively (SPSS for Windows, Inc., Chicago, IL, USA). There were no missing data. In both trials, normality of data was assessed using histograms with normal curves and Shapiro‒Wilk tests (with significance set at *p* = 0.05). Data were judged to be normally distributed; therefore, parametric statistics were conducted. Pre- and post- outcome data for the STAI-AD were analysed using 2-way mixed factorial analyses of variance (ANOVA). Paired t tests were used to investigate any significant interaction effects. Age and gender were not included as potentially moderating variables in the analyses as groups were random samples with the aim of the trials being to examine the health outcomes of the bouts of walking in both genders and in a wide age range (18–65). Partial eta squared (η^2^) was used to estimate the effect size for each *F*-test, and was set as 0.1 (small), 0.4 (medium) and 0.8 (large) (Richardson, 2011). Cohen's *d* was used to estimate effect sizes for pre-post data by group in both trials, and was set as 0.2 (small), 0.5 (medium) and 0.8 (large) (Cohen, 1988). This allowed further interpretation of the data and to avoid drawing conclusions solely on *p*-values (Wasserstein et al., 2019). Independent samples t tests, with Cohen's *d* were conducted to examine post-walking, between-groups differences in RPE. Statistical significance was defined as *p* < 0.05 for all tests with data presented as mean (*M*) and standard deviation (*SD*) unless otherwise stated.

## Results

### Trial 1 - participants

**Figure 2.**
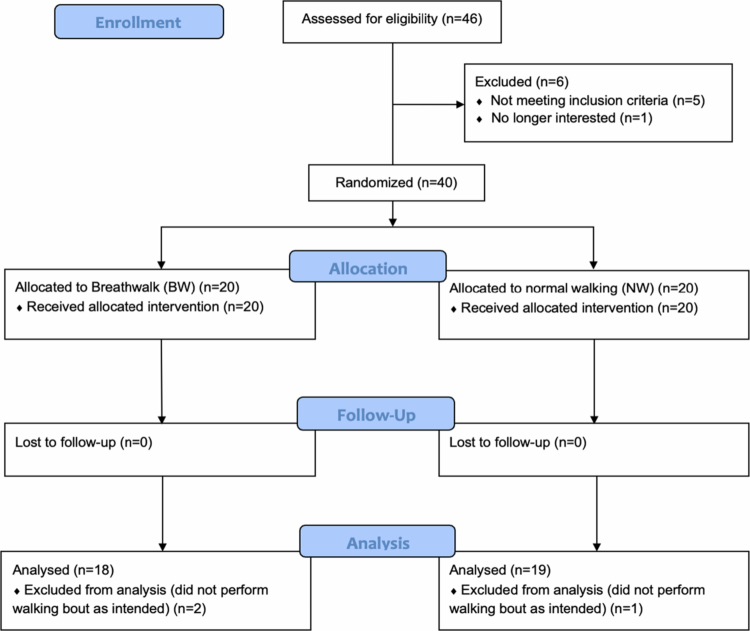
CONSORT flow of participants through Trial 1.

From 46 initial responses, 40 participants met inclusion criteria with 37 individuals (27 females, 10 males, mean age ± *SD* 38.54 ± 10.66) ultimately analysed (BW group, *n* = 18, 11 females, 7 males; NW group, *n* = 19 participants, 16 females, 3 males). Data collection took place between June and July 2018. The flow of participants is shown in Figure 2. Table 3 displays descriptive statistics for all outcome measures.

**Table 3.** Descriptive statistics for pre- and post-outcomes (anxiety and RPE). Values are presented as mean (M) with standard deviation (*SD*).

Parameter	BW group (*n* = 18)		NW group (*n* = 19)	
	Pre	Post	Pre	Post
STAI-AD	27.9 (4.77)	25.11 (5.53)	32.32 (5.65)	30.53 (5.06)
RPE^*^	n/a	9.3 (1.8)	n/a	9.5 (2.0)

STAI-AD – State-Trait Anxiety Inventory Questionnaire for Adults™; PRE – Rating of Perceived Exertion Scale *Only assessed post-intervention.

### Trial 1 – outcome analysis

#### Anxiety (STAI-AD)

There was no significant interaction effect between NW and BW groups and time (*p* = 0.529; η^2^ = 0.011). There was a significant main effect of time on anxiety level, *F* (1, 35) = 7.9 (*p* = 0.008; η^2^ = 0.184) indicating a small-medium reduction in anxiety over time for all participants. However, Cohen's *d* calculations highlighted a larger effect size for pre-post change for the BW group (*d* = 0.63) compared with the NW group (*d* = 0.33).

#### Rating of perceived exertion scale (RPE)

Mean RPE post-trial was not statistically different between the groups *t*(35) = −0.398, *p* = .692. However, the difference did represent a small effect size (*d* = 0.13) in favour of the BW group (*M* = 9.28, *SD* = 1.84) which had a lower mean RPE compared with the NW group (*M* = 9.53, *SD* = 1.95).

### Trial 2 – participants

**Figure 3.**
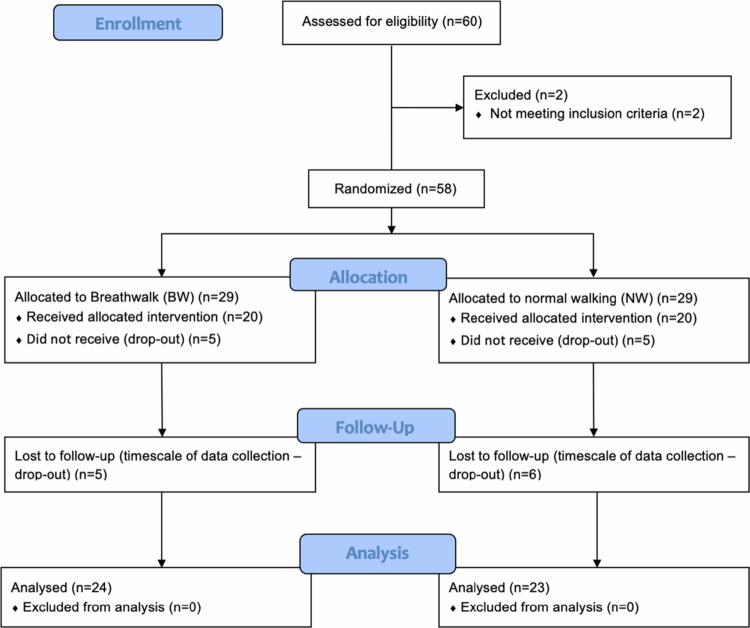
CONSORT flow of participants through Trial 2.

From 60 initial responses, 47 participants (36 females, 11 males, 20.96 ± 1.56 years, mean walk time 14.23 ± 5.04 min) were randomised and provided complete data for analysis; BW group (*n* = 24 participants, 19 females and 5 males) and NW group (*n* = 23 participants, 17 females and 6 males). Data collection took place between January and February 2020. The flow of participants is shown in Figure 3. Table 4 displays descriptive statistics for all outcome measures.

**Table 4.** Descriptive statistics for pre- and post-outcomes (anxiety and RPE). Values are presented as mean (M) with standard deviation (*SD*).

Parameter	BW group (*n* = 24)		NW group (*n* = 23)	
	Pre	Post	Pre	Post
STAI-AD	33.4 (7.8)	28.0 (4.3)	31.9 (7.5)	30.3 (8.1)
RPE^*^	n/a	8.5 (1.4)	n/a	8.8 (1.9)

STAI-AD – State-Trait Anxiety Inventory Questionnaire for Adults™; PRE – Rating of Perceived Exertion Scale *Only assessed post-intervention.

### Trial 2 – outcome analysis

#### Anxiety (STAI-AD)

A significant interaction effect was identified between group (BW, NW) and time (pre, post) in terms of state anxiety (*F* (1, 45) = 4.579, *p* = .038, medium-to-large effect *η*^2^ = 0.092). Paired t-tests revealed there was a large, significant decrease in anxiety pre- to post-walk in the BW group, *t*(23) = 4.17, *p* < .001, *d* = 0.85, but a non-significant decrease (with a small effect) for the NW group, *t*(22) = 1.28, *p* = .214, *d* = 0.27).

#### Rate of perceived exertion

Mean RPE post-trial was not statistically different between the groups *t*(45) = −0.101, *p* = .920. However, the difference did represent a small effect size (*d* = 0.22) in favour of the BW group (*M* = 8.5, *SD* = 1.4) which had a lower mean RPE compared with the NW group (*M* = 8.8, *SD* = 1.8).

## Discussion

### Principal findings

Findings from these two pilot randomised studies provide preliminary evidence that taking a short bout of free-living moderate intensity walking whilst performing a breathwalk produces additional anxiolytic benefits than normal walking. The simple addition of performing slow, deep and regulated breathing whilst walking for a short every day journey led to a statistically significant finding in one trial, and large effect sizes in both trials. To the best of our knowledge this study is the first to empirically examine the purported mental health benefits of performing the breathwalk technique in healthy adults. The necessity of this study stems from the increasing mental health burden, particularly since COVID-19, and the identified need to develop interventions to help manage anxiety disorders (Santomauro et al., 2021). The use of these findings has the potential to optimise the effectiveness of walking interventions to enhance mental health and well-being.

### Comparison with previous literature

Our findings further support previous research, including findings from multiple meta-analyses (Connor et al., 2023; Ensari et al., 2015; Petruzzello et al., 1991), which have demonstrated that acute bouts of PA, in this case walking, can reduce state anxiety in adult populations with small effects evident. These findings contribute to the limited evidence base on walking (Kelly et al., 2018) and also demonstrate that moderate-intensity activity durations of less than 30 min can be beneficial for state anxiety. This finding is important because it is known that shorter bouts of activity are likely to be more easily integrated into daily activity patterns, and walking is a mode of physical activity that aligns with this approach whilst being attractive and accessible to almost all of the population (Bull & Hardman, 2018).

Our findings expand on previous work by indicating that incorporating a yoga-based breathing protocol (BW) to short-bouts of walking can lead to larger reductions in anxiety levels in comparison to those who performed normal walking. These findings support previous research (Edwards et al., 2018; Shin et al., 2013) which has demonstrated the additional benefits of meditation on walking. Further research is needed to gain a better understanding of the mechanisms underpinning the anxiolytic effects of exercise (Connor et al., 2023). Nevertheless, Edwards and colleagues proposed that the distraction hypothesis may explain the additional benefits of meditation, as the activity can deflect attention from external stressors such as anxious or depressive thoughts (Edwards et al., 2018). This hypothesis also has relevance to the current findings, where the focus on regulated breathing during walking may have provided a distraction from external stressors. A further potential mechanism for this beneficial effect may be the triggering of lower arousal emotions through meditation, which are associated with lower levels of anxiety, in comparison to increases in arousal induced by exercise (Jones et al., 2018).

### Implications for practice

BW differs to other yoga or meditation-style breathing techniques, and at present comparisons between the effectiveness of different techniques are so far undetermined. However, BW has advantages due to its simplicity, and strict parameters making it easy to explain and implement and for participants to remember and perform. Whilst the findings of this study are tentative in nature, they are positive and provide justification for the investigation of the BW technique on a wider scale, in different populations and with different health outcomes. If these initial findings are supported then the incorporation of breathwalk into walking programmes and promotion activities could lead to meaningful reductions in anxiety within the general public. As an example, aligned with ISPAH's eight best investments (Milton et al., 2021), recent evidence suggests that within communication campaigns messages should ‘highlight short-term outcomes (specifically relating to social and mental health’ (Williamson et al., 2020, *p*. 8). Whilst not investigated specifically as a variable in this study, using BW during student's active travel rather than leisure-time PA for anxiety reduction may be an important factor. Anxiety disrupts cognitive performance (Maloney et al., 2014), suggesting the morning active commute to school or work may be an important domain to target in order to positively influence subsequent productivity during the day (Ma & Ye, 2019).

### Strengths and limitations

A strength of these pilot trials was the randomised designs, enhancing internal validity and minimising bias. Conducting multiple trials across domains of walking and in different settings also enhances ecological validity and allows for the reproducibility of the acute effects of the intervention to be evident. However, there were several limitations. In both trials, the necessary instruction of the BW technique made assessor and subject blinding impractical, potentially leading to performance bias and reducing internal validity. Lack of blinding has been identified as a recurring issue in studies of this kind (Connor et al., 2023). Convenience sampling may have led to unintentional selection bias in recruiting participants from the researchers' networks who were potentially more physically active than the general population. Additionally, simple randomisation methods meant that there was no stratification to intervention arm by gender, age or other sociodemographic characteristic. These aspects limit the generalisability of findings (Jager et al., 2017). Additionally, baseline anxiety levels were not considered in recruitment and with participants scoring less than 34, there is potentially a floor effect as evident in other similar studies (Connor et al., 2023). It is likely that the limitations have some impact on the findings, and thus future research could consider replicating these studies with more homogenous samples to explore moderating factors and potentially differential effects for example, by gender groups, or those with high/low levels of state anxiety or levels of physical activity at baseline.

Relatively small sample sizes, exacerbated by participant drop-out and unavailability, may have decreased the ability to detect significant differences in Trial 1, although an alternative hypothesis would be that a longer walking bout (such as that in Trial 2) is required for the additional benefit of breathwalk to become evident. There were also several potential confounding factors in the two trials that were not accounted for in the design or analyses. For example, in Trial 2, the external environment of the chosen route to university could potentially affect the psychological well-being of participants. The participants' commuting routes varied; some were exclusively urban, whereas others incorporated green space. (Song et al., 2015) found significantly lower anxiety scores after an urban park walk than after a city area walk. Additional potential moderating factors include baseline levels of physical activity or state anxiety and time of day of the walking bout, which should all be assessed in future trials. However, it should be noted that these studies were not designed to test specific hypotheses (Eldridge et al., 2016); therefore, caution must be given to interpreting these findings purely on statistical significance particularly given recent concerns raised on this topic (Wasserstein et al., 2019). The calculation of effect sizes offers an additional level of interpretation and support that the breathwalk technique is worthy of further investigation through larger-scale testing. Critical to future trials is the incorporation of collecting process evaluation data to explore participants' experiences of walking using the breathwalk protocol.

## Conclusion

The presented trials provide further evidence that short, single bouts of walking improve state anxiety in healthy young adults, and that these benefits can be achieved in different environments and for different purposes. There were indications that the incorporation of a simple, easy-to-use breathing technique (breathwalk) provides greater anxiolytic effects than walking alone. These findings provide support for the proof of concept and subsequent research is warranted. Future studies should be conducted with larger sample sizes across a broader range of outcome measures involving different population groups.

## Supplementary Material

Additional File 3 TIDieR checklist.docx Additional File 3 TIDieR checklist.docx

Additional file 1_CONSORT extension for Pilot and Feasibility Trials Abstracts Checklist.doc Additional file 1_CONSORT extension for Pilot and Feasibility Trials Abstracts Checklist.doc

Additional file 2_CONSORT extension for Pilot and Feasibility Trials Checklist.doc Additional file 2_CONSORT extension for Pilot and Feasibility Trials Checklist.doc

## Data Availability

The datasets produced in the current study are available from the corresponding author on reasonable request.
