# Supplementary material for: Examining the impact of combining yoga-based breathing techniques with short-bouts of walking on state anxiety: findings from two pilot randomised trials
Source: Health Psychol Behav Med. 2026 Feb 13;14(1):2629646. doi: 10.1080/21642850.2026.2629646 (PMC12912209; doi:10.1080/21642850.2026.2629646)
Supplement: Additional file 1_CONSORT extension for Pilot and Feasibility Trials Abstracts Checklist.doc [file RHPB_A_2629646_SM7576.doc]

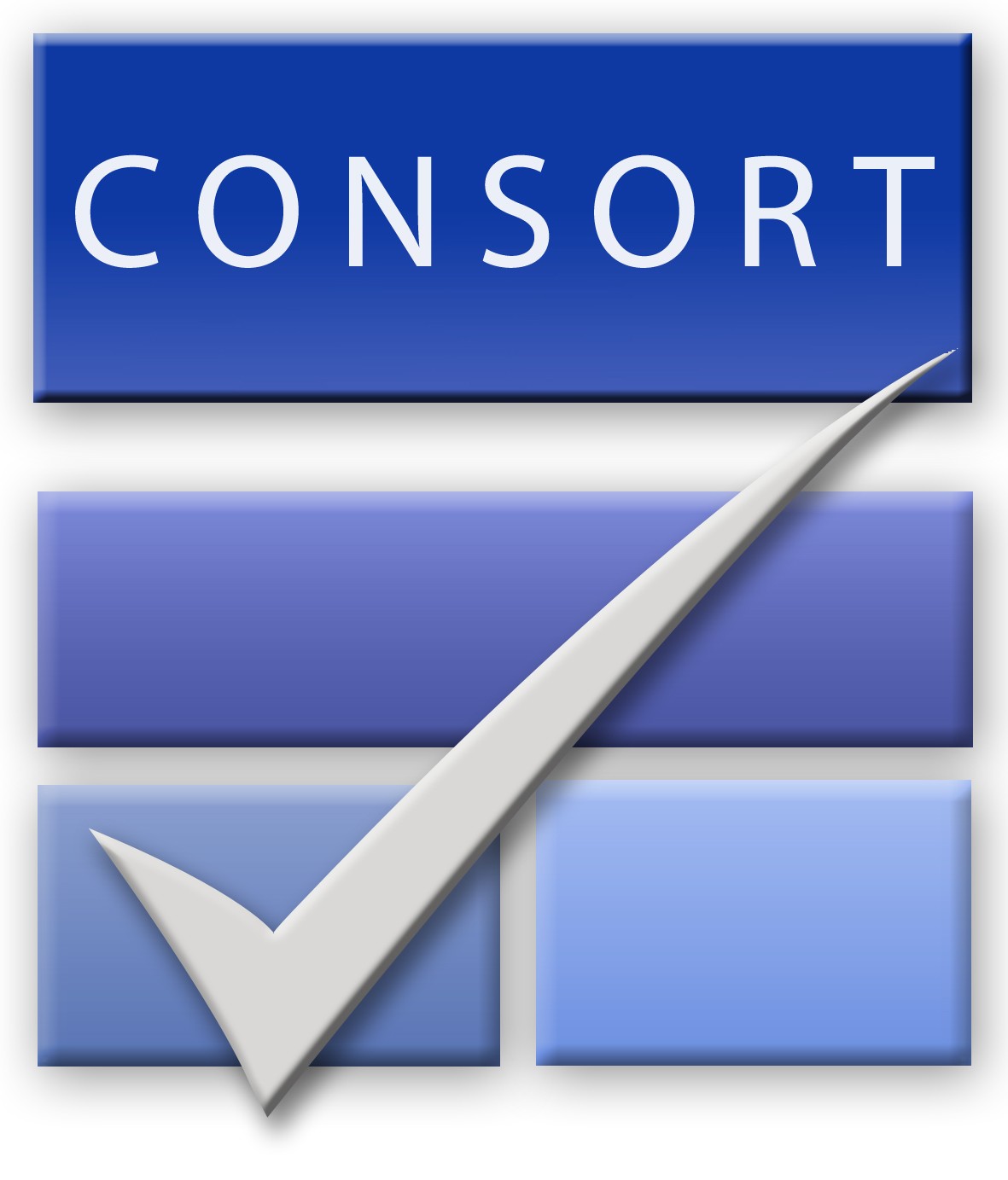
**CONSORT 2010 checklist of information to include when reporting a pilot or feasibility randomized trial in a journal or conference abstract**

| **Item** | **Description** | **Reported on line number** |
| --- | --- | --- |
| Title | Identification of study as randomised pilot or feasibility trial | See title page |
| Authors * | Contact details for the corresponding author | NA |
| Trial design | Description of pilot trial design (eg, parallel, cluster) | See methods |
| Methods |  |  |
| Participants | Eligibility criteria for participants and the settings where the pilot trial was conducted | See methods |
| Interventions | Interventions intended for each group | See methods |
| Objective | Specific objectives of the pilot trial | See aim in background |
| Outcome | Prespecified assessment or measurement to address the pilot trial objectives** | See methods |
| Randomization | How participants were allocated to interventions | See methods |
| Blinding (masking) | Whether or not participants, care givers, and those assessing the outcomes were blinded to group assignment | See methods |
| Results |  |  |
| Numbers randomized | Number of participants screened and randomised to each group for the pilot trial objectives** | See Figure 1 |
| Recruitment | Trial status† | NA |
| Numbers analysed | Number of participants analysed in each group for the pilot objectives** | See Figure 1 |
| Outcome | Results for the pilot objectives, including any expressions of uncertainty** | See results |
| Harms | Important adverse events or side effects | Outcome not  reported |
| Conclusions | General interpretation of the results of pilot trial and their implications for the future definitive trial | See discussion |
| Trial registration | Registration number for pilot trial and name of trial register | Trial not registered (no funds for registration costs) |
| Funding | Source of funding for pilot trial | No funding reported |

Citation: Eldridge SM, Chan CL, Campbell MJ, Bond CM, Hopewell S, Thabane L, et al. CONSORT 2010 statement: extension to randomised pilot and feasibility trials. BMJ. 2016;355.

**this item is specific to conference abstracts*

***Space permitting, list all pilot trial objectives and give the results for each. Otherwise, report those that are a priori agreed as the most important to the decision to proceed with the future*

*definitive RCT.*

*†For conference abstracts.*
